# Long-term stability of return to work after a workplace-oriented intervention for patients on sick leave for burnout

**DOI:** 10.1186/1471-2458-14-821

**Published:** 2014-08-09

**Authors:** Björn Karlson, Peter Jönsson, Kai Österberg

**Affiliations:** Department of Psychology, Lund University, Box 213, SE 221 00 Lund, SWEDEN; Division of Occupational and Environmental Medicine, Lund University, SE 221 85 Lund, Sweden; School of Education and Environment, Section of Psychology, Kristianstad University, SE 291 88 Kristianstad, Sweden

**Keywords:** Sick leave, Return to work, Burnout, Exhaustion disorder, Workplace intervention, Follow-up

## Abstract

**Background:**

The period from the mid-1990s to the mid-2000s saw a rapid increase in long-term sick leave in Sweden, primarily due to mental illness and often related to job burnout. This led to an urge for effective treatment programs that could prevent the often long sick leaves. In 2010 we presented a newly developed work-place intervention method, showing that 89% of the intervention group had returned to work at a 1.5 year follow-up, compared to 73% of the control group. The main aim of this study was to assess the long-term stability of these promising results.

**Methods:**

Sick leave registry data from the Regional Social Insurance Office were analyzed for an additional year (50 weeks) beyond the original 1.5 year period (80 weeks). Data from 68 matched pairs of intervention participants (IP) and controls were available. The proportions of participants being on full-time sick leave versus having returned to work to any extent were computed for every 10^th^ week. Generalized estimating equations were used with GROUP (IP versus controls) as between-subjects factor, WEEKS and AGE as covariates, and return-to-work (RTW) as dependent variable. Significant differences (Wald χ^2^ with α ≤ .05) was followed up with polynomial contrasts. Individual relapses to higher degrees of sick leave (e.g. from 50% to 100%) and whether partial RTW led to later full-time RTW, were also analyzed.

**Results:**

The omnibus test over all 130 weeks showed a GROUP*WEEKS interaction effect (*p* = .02), indicating differential group developments in RTW, though similarly high at week 130 in both groups with 82.4% of the IP and 77.9% of the controls having RTW (*p* = .22; χ^2^-test). A significant interaction with age led to separate analyses of the younger and older subgroups, indicating a stable pattern of superior RTW only among younger IP (week 130: 88.6% vs. 69.7%, *p* = .054; χ^2^-test). There was no group difference in relapses into increased degree of sick leave. Part-time sick leave did not predict a later stable full-time RTW.

**Conclusions:**

The previously reported improvement in RTW with the newly developed workplace-oriented intervention showed a long-term stability only among younger participants.

## Background

The period from the mid-1990s to about the mid-2000s saw a rapid increase in long-term sick leave in Sweden. This sick leave increase was primarily due to mental illness, which often appeared to be related to long-term work stress and exhaustion. Periods of sick leave were often very long and could even lead to disability pension or loss of employment. Although the number of persons on long-term sick leave has decreased during recent years, the proportion of people with a mental illness diagnosis remains at about thirty percent [1].

Sick leave due to exhaustion disorder (ED) is a Swedish diagnosis similar to what has been labeled clinical burnout in many other countries. It is important to treat ED adequately if the aims are to facilitate return to work (RTW). However, in previous studies treatments aimed at supporting and developing individual coping strategies – treatments commonly based on the framework of cognitive behavioral therapy – have not shown any significant impact on RTW [2–6]. On the other hand, workplace-oriented interventions, aimed at changing the person’s work situation, have largely shown promising results, though there are few such previous studies on this patient group [7–9]. One study compared RTW in two groups of women on sick leave for stress-related diagnoses. One group was part of an intervention labeled Redesigning Daily Occupations (ReDO) and the other group received care as usual (CAU). The results showed an improvement in RTW in the intervention group compared to the CAU group, which showed no improvement [10].

However, there are additional indirect indications of the superiority of interventions involving the workplace. Let us consider musculoskeletal conditions, an area in which more intervention studies have been carried out. A recent review analyzed whether interventions aimed at musculoskeletal conditions that involved the workplace had a better effect on RTW than did interventions not involving the workplace. It found that workplace involvement in general had no effects, but that interventions including consultation and consensus between stakeholders (i.e., the employee, the workplace and occupational health professionals), and subsequent work modification, did have positive effects on RTW [11]. In line with this, van Oostrom et al. [12] drew upon an existing successful participatory intervention protocol for low back pain when they designed a similar protocol for stress-related mental disorders. Some core components of these protocols were identification of barriers to and solutions for RTW in a process led by an RTW coordinator, which was followed by conversations between the sick-listed employee and his/her supervisor, as well as separate discussions with the employee and the employer. In an initial randomized RTW study of stress-related mental disorder, no overall effect of the intervention was found. However, employees who at baseline had expressed an intention to return to work despite symptoms benefited from the intervention [9].

Van Oostrom’s model [9] seems to have many characteristics in common with the structured workplace-oriented intervention model that has been developed by our research group and that aims at facilitating RTW in persons on long-term sick leave due to work-related burnout [8]. One key component of our model was a team-supported patient-supervisor dialogue that concerned how to achieve sustainable work resumption, including agreements on necessary changes in working conditions. The model was tested as a controlled clinical trial with inclusion of participants during a 3-year period (2003 – 2006), followed by an approximate 1½-year follow-up of RTW. The intervention group showed a superior rate of RTW compared to a control group that only received CAU, with no specific intervention (89% vs. 73%, respectively, back to work to some extent) [8]. The rate of return to full-time work was equal in the two groups, but in the intervention group many had returned on a part-time basis, while a significantly larger proportion of the untreated control group was still on full-time sick leave.

A later follow-up of a subsample of the intervention group, unrelated to the RTW study cited above and with a neuropsychological focus, showed that self-ratings of exhaustion, anxiety and depressive symptoms and subjective cognitive complaints had decreased substantially after a (mean) 20-month period [13], but were still markedly higher at follow-up than among healthy referents [14]. Although previous studies have shown that RTW is possible despite symptoms [2, 7], such residual symptoms may constitute a certain degree of vulnerability, implying a risk for relapse into sick leave upon exposure to increased work stress.

Most rehabilitation studies on sick leave due to burnout have employed a rather short time to follow-up. Thus, we have less knowledge of longer-term trajectories of post-intervention work ability. One of the few such investigations is a prolonged follow-up study three years after completion of an intervention comprising 1-year-long treatments with either Qigong combined with cognitive behavioral rehabilitation or Qigong only. After the 3-year follow-up, RTW rates from the original 1-year follow-up improved from about 60% to about 75% in both groups. However, those who had received the combined treatment had reduced burnout symptoms, were taking less anti-depressive medication, and had increased their use of cognitive tools learned in the program [15]. In another study, a small group from a comparable patient category, who had received individual-oriented rehabilitation, showed a similar increase to 40% gainful employment in the intervention group as well as in the control group at a 5-year follow-up [5].

The follow-up period in our study was also rather short (approximately 18 months), and there are a number of reasons for conducting a prolonged follow-up of the stability of RTW. The two studies cited above showed improvements in RTW rate over a longer time span, however, this was independent of the intervention. Our intervention, in contrast, mainly focused on changes at the workplace. It involved not only planning and agreement concerning short-term strategies to facilitate RTW, but also concerning long-term strategies and actions thought to be important for sustainable work ability and work life participation. To our knowledge, no long-term follow-ups of the few studies of that kind have been made. Although an investigation of the actual fulfillment of plans and agreements is beyond the scope of the present study, the main outcome measure, i.e. RTW, will be analyzed. Because the RTW rate was already high at the first follow-up, there might be a slight ceiling effect for RTW, particularly in the intervention group. However, considering the lack of group differences in the two cited studies, it is possible that our control group as well will show a RTW rate similar to that of the intervention group at a prolonged follow-up, but with a certain delay. On the other hand, considering possible vulnerability due to residual symptoms, as mentioned above, there may be relapses into new long-term sick leave in one or both of the groups. Thus, one main question concerns the sustainability of RTW. It has also been suggested that part-time RTW could be a pathway to full-time work [7] – an interesting assumption that requires further examination. In contrast, another possibility is that part-time work is the most some individuals can manage after recovering from burnout.

The present aims are to study (a) whether the superior RTW after a specific workplace-oriented intervention for persons on sick leave due to burnout was sustained or increased further during an additional twelve months, or whether the intervention merely speeded up the course of RTW, if so indicated by a converging RTW rate between the two groups during the extended follow-up. Supplementary aims were to study (b) whether relapses into increased degree of sick leave were less frequent in the intervention group, and (c) whether initial part-time RTW could predict a successful full-time RTW.

## Methods

### Procedures

The procedures for patient selection, clinical examination, intervention and collection of register data on sick listing have previously been described in detail [8]. In brief, the participants were recruited in cooperation with the regional social insurance offices (RSIO), which provided consecutive new sick-listed cases for the period 2003–2006. Basic inclusion criteria were employment, sick-listing at least half-time for 2–6 months following a previously healthy state, and having an International Classification of Disease (ICD-10) diagnosis within the F43 category (with the exception of post-traumatic stress disorder) due predominantly, although not necessarily exclusively, to work-related stressors, with the exception of severe conflicts or bullying. Those who fulfilled the criteria, accepted participation, and completed the entire program were included in the analyses as the intervention group (IG). The program involved responding to a number of questionnaires, being examined by a multi-disciplinary team, one member of which also interviewed the patient’s supervisor, and finally participating in the intervention involving a team-supported dialogue (the Convergence Dialogue Meeting; CDM) between the patient and his/her supervisor. The control group (CG) was chosen from those who were invited to participate but were not interested, without giving any specific reason for their disinterest. They were randomly selected from persons who matched the IG in terms of age, and degree and duration of sick leave at the time of the CDM. In contrast to the IG, the CG was only followed by examining the RSIO’s register data on sick listing.

### Participants

The 74 individuals with clinical burnout (Swedish diagnosis: Exhaustion Disorder) who participated in our first intervention study (2003–2006), together with 74 untreated controls, were sent a letter asking whether they would allow us to access their sick-listing data once again to examine RTW for subsequent years. At this time, six subjects (two from the IG and four from the CG) denied us use of their data. In addition three subjects (one from the IG and two from the CG) had retired from work. Due to the fact that individual matching of IG and CG participants in pairs had been used in our initial intervention study, the loss of nine persons would have resulted in 9 lost pairs (18 lost participants), which would have reduced the power of the study. To decrease participant loss, the original matching for the 9 + 9 persons was broken up, and the remaining participant in each broken-up pair was re-matched with a participant among those who would otherwise have been lost, in case an appropriate new matching based on the original criteria could be accomplished. In step one, IG participants from broken pairs were re-matched with otherwise lost controls and in step two the CG participants from broken pairs were similarly matched with remaining unmatched IG participants. The procedure resulted in three successful re-matched pairs, reducing the total drop-out to 6 pairs, providing 68 matched pairs available for analyses. In the entire group, including 110 (81%) women, the mean as well as median age, at their first day of sick leave, was 45.5 years; the range was 25–62 years. The drop-out group consisted of 5 women and 7 men, with a median age of 58 years at their first day of sick leave. At the end of the previous follow-up period (week 80) 42% of the drop-outs had full RTW, 42% were on part-time sick leave and 17% were on full-time sick leave. The clinical condition of the IG participants at the baseline examination some months before the CDM have been described in detail in our previous paper (Table 1 in Karlson et al. [8]), and the drop-out of six IG participants did not change the clinical group characteristics to any appreciable extent.

**Table 1:** **Sick leave in the older halves of the intervention and control groups as a function of weeks**

	Sick leave (%)					χ ^2^	***P***
	0	25	50	75	100		
	Week 0						
Control*	14.3 (5)	5.7 (2)	34.3 (12)	0	45.7 (16)	6.08	.193
IG**	9.1 (3)	12.1 (4)	33.3 (11)	12.1 (4)	33.3 (11)		
	Week 10						
Control	34.3 (12)	17.1 (6)	14.3 (5)	2.9 (1)	31.4 (11)	5.83	.212
IG	18.2 (6)	9.1 (3)	36.4 (12)	3.0 (1)	33.3 (11)		
	Week 20						
Control	48.6 (17)	8.6 (3)	14.3 (5)	0	28.6 (10)	7.29	.063
IG	18.2 (6)	18.2 (6)	24.2 (8)	0	39.4 (13)		
	Week 30						
Control	45.7 (16)	11.4 (4)	11.4 (4)	0	31.4 (11)	5.34	.254
IG	30.3 (10)	21.2 (7)	9.1 (3)	9.1 (3)	30.3 (10)		
	Week 40						
Control	48.6 (17)	8.6 (3)	11.4 (4)	2.9 (1)	28.6 (10)	1.81	.771
IG	39.4 (13)	18.2 (6)	15.2 (5)	3.0 (1)	24.2 (8)		
	Week 50						
Control	54.3 (19)	8.6 (3)	11.4 (4)	2.9 (1)	22.9 (8)	2.20	.699
IG	45.5 (15)	15.2 (5)	12.1 (4)	9.1 (3)	18.2 (6)		
	Week 60						
Control	65.7 (23)	8.6 (3)	5.7 (2)	2.9 (1)	17.1 (6)	3.01	.556
IG	48.5 (16)	12.1 (4)	12.1 (4)	9.1 (3)	18.2 (6)		
	Week 70						
Control	68.6 (24)	5.7 (2)	5.7 (2)	0	20.0 (7)	2.67	.445
IG	51.5 (17)	6.1 (2)	15.2 (5)	0	27.3 (9)		
	Week 80						
Control	68.6 (24)	5.7 (2)	2.9 (1)	0	22.9 (8)	4.38	.223
IG	57.6 (19)	6.1 (2)	18.2 (6)	0	18.2 (6)		
	Week 90						
Control	68.6 (24)	8.6 (3)	5.7 (2)	0	17.1 (6)	2.09	.555
IG	57.6 (19)	6.1 (2)	15.2 (5)	0	19.1 (13)		
	Week 100						
Control	68.6 (24)	8.6 (3)	5.7 (2)	0	17.1 (6)	2.19	.701
IG	57.6 (19)	9.1 (3)	12.1 (4)	3.0 (1)	18.2 (6)		
	Week 110						
Control	71.4 (25)	5.7 (2)	2.9 (1)	0	20.0 (7)	4.63	.328
IG	54.5 (18)	12.1 (4)	12.1 (4)	3.0 (1)	18.2 (6)		
	Week 120						
Control	74.3 (26)	2.9 (1)	5.7 (2)	0	17.1 (6)	3.31	.000
IG	57.6 (19)	9.1 (3)	9.1 (3)	3.0 (1)	21.2 (7)		
	Week 130						
Control	77.1 (27)	0	8.6 (3)	0	14.3 (5)	4.94	.176
IG	54.5 (18)	6.1 (2)	15.2 (5)	0	24.2 (8)		

*n = 35.

**n = 33.

The participants were 46 years or older at their first day of sick leave. Figures show percentages within each group and the corresponding number of participants. Group comparisons with Pearson χ2-test were done on the full range of sick leave steps.

### Data sampling methods

In the original follow-up, sick-leave data for 80 consecutive weeks after the CDM were used. The data were collected from the sick leave registers at the RSIO until August 2009. In the present prolonged follow-up, similar data were collected until August 2011, i.e. in total 183 weeks. However, in the analyses 130 weeks were used, i.e. approximately one additional year from the end of the previous 80-week follow-up period. There were two main reasons for this limitation on data usage: (1) there was a change in the social insurance system from January 2011 that was intended to reduce the time an individual could be on sick leave, though we can assume adaptations to the change were already initiated some months before that date, and (2) due to age retirement, extending the follow-up period an additional year would have further reduced the group sizes.

The degree of sick leave (0, 25, 50, 75, or 100% of ordinary working time) for each week was entered as variables (i.e., week x: 50%, week y: 75%, etc.). A week was defined as four days or more [8].

### Statistical analyses

The main analysis of the development of RTW were based on dichotomized sick leave data, that is, RTW 25% or more (YES) vs. not back to work (NO), using generalized estimating equations (IBM SPSS Statistics 21.0) to examine omnibus effects with GROUP (IG and CG) as a between-subjects factor and WEEKS (every 10th week from W0 to W130, i.e. 14 time points) and AGE as covariates, with RTW as the dependent variable. Logit link function and auto regressive (AR (1)) correlation matrix were used. Significance testing was performed using Wald χ^2^ (α = .05). Significant effects were followed up with polynomial contrasts.

Point wise analyses of RTW on separate weeks were carried out with Pearson χ^2^-test, on either the group distributions of the full range of sick leave steps (0, 25, 50, 75, or 100%) or on dichotomized sick leave data, that is, RTW ≥25% vs. no RTW (i.e. 100% sick leave).

Number of relapses into an increased degree of sick leave (e.g. from 25% sick leave to 50%, or from full RTW to 25% sick leave) was analyzed by counting all periods of at least two consecutive weeks with increased sick leave compared to the preceding week, for each participant. Group distributions were compared with Pearson χ^2^-test (α = .05).

Whether initial part-time sick leave could predict later full-time RTW was analyzed by comparing the implementation of part-time sick leave among those having attained a stable full time RTW during the final six months (26 weeks) of the follow-up period versus those not fulfilling this criterion. Implementation of part-time sick-leave was defined as either (a) at least four weeks of part-time sick-leave during the preceding 104 weeks or (b) at least 6 months (26 weeks) of part-time sick-leave during the same period. Group frequency distributions were compared with χ^2^-test and Mann–Whitney U test (α = .05).

### Ethics

All participants were informed about the purpose of the study and were given the possibility to deny us use of their sick-listing register data. The study protocol was approved by the Regional Ethical Review Board of Lund University (Reg. no. 2011/145).

## Results

### General development of RTW

At the end of the extended follow-up period (week 130) 82.4% of IG and 77.9% of the CG participants were back at work to any extent, a difference that was not statistically different (*p* = .22, dichotomized data). The omnibus test over all 130 weeks showed a significant GROUP*WEEKS interaction effect: χ^2^(1) = 5.50, *p* = .019, β = 0.474, 95% CI = 0.078 – 0.871, indicating a differential development in RTW across groups. However, also a GROUP*WEEKS*AGE interaction was found: χ^2^(1) = 5.18, *p* = .023, β = -.01, 95% CI = -.019 – (- .001). To examine the effect of AGE, the group was mean split by age 45.5 years (*n* = 68 per group), and the resulting age groups were analyzed separately.

**Figure 1:**
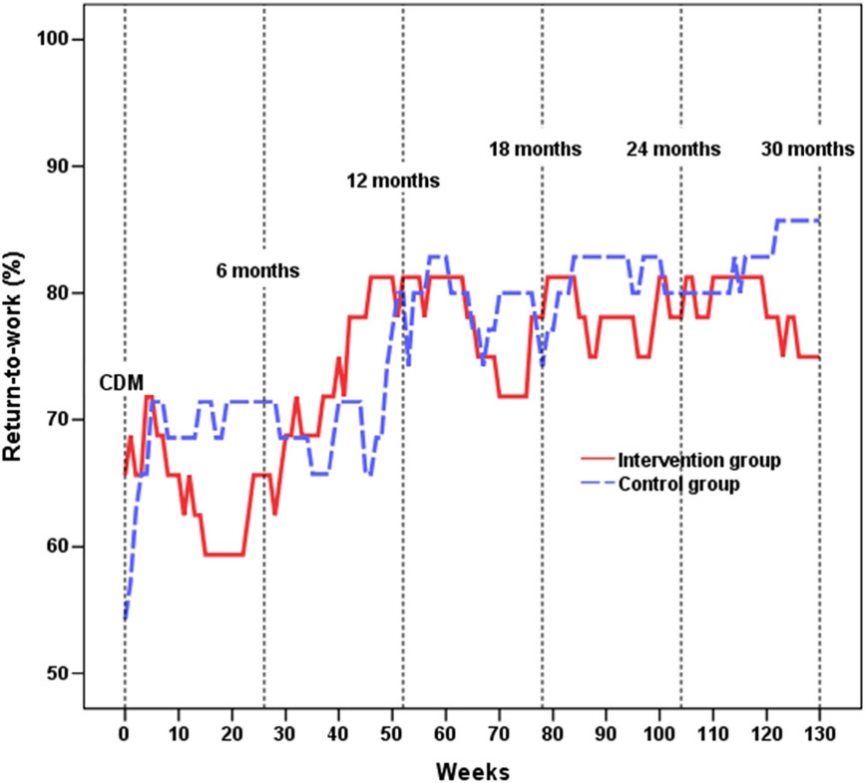
**Rates of return-to-work on at least 25% of full time among older participants.** The older halves of the groups were 46 years or older on their first day of sick leave. Values for the Intervention Group are shown with a solid red line, and for the Control Group with a dashed blue line, from the time of the Convergence Dialog Meeting (CDM) until Week 130.

Considering the group with older individuals, a main effect of WEEKS was found: χ^2^(1) = 9.29, *p* = .002, β = 0.128, 95% CI = 0.050 – 0.205. No other significant effects were found. Polynomial contrasts showed that the number of individuals returning to work increased over time [linear contrast: χ^2^(1) = 10.75, *p* = .001], but stabilized after about 60 weeks [quadratic contrast: χ^2^(1) = 4.58, *p* = .032]; see Figure 1 and Table 1. In week 130, 75.8% of the IG and 85.7% of the CG participants were back at work to any extent, a difference that was not statistically significant (*p* = .30, dichotomized data). Since RTW was, on a whole, rather similar in the older groups over the entire follow-up period, no signs of converging RTW between groups could be detected (Figure 1).

**Figure 2:**
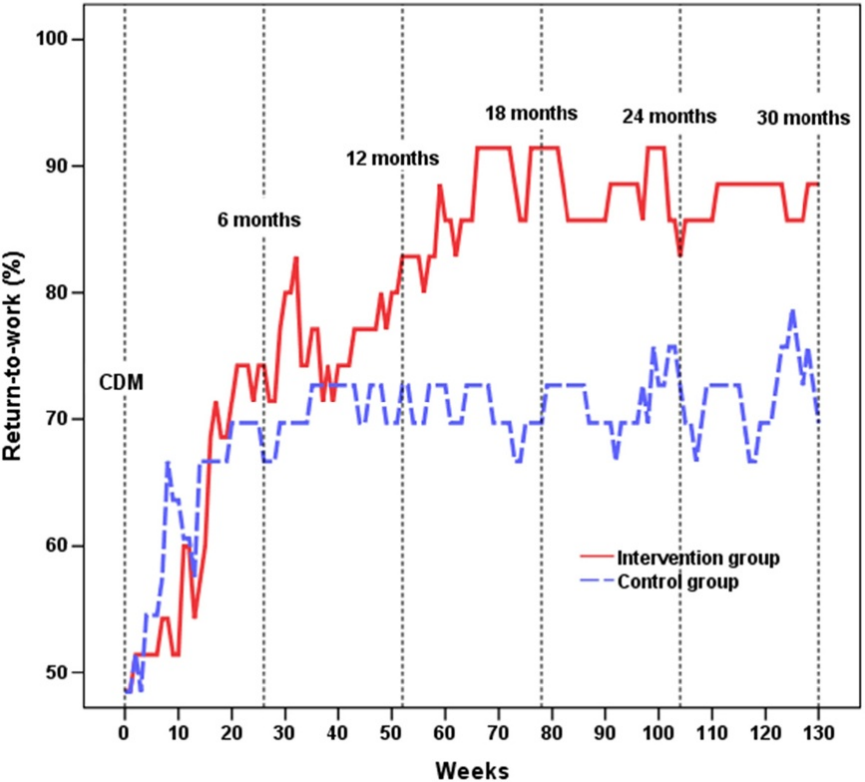
**Rates of return-to-work on at least 25% of full time among younger participants.** The younger halves of the groups were 45 years or younger on their first day of sick leave. Values for the Intervention Group are shown with a solid red line, and for the Control Group with a dashed blue line, from the time of the Convergence Dialog Meeting (CDM) until Week 130.

Like in the older group, a main effect of WEEKS was found for the younger group: χ^2^(1) = 16.73, *p* < .0001, β = 0.062, 95% CI = 0.000 – 0.124. However, a GROUP*WEEKS interaction was also revealed: χ^2^(1) = 4.47, *p* = .034, β = 0.133, 95% CI = 0.010 – 0.256. The number of individuals in the IG having returned to work increased until week 70, as indicated by a linear contrast [χ^2^(1) = 13.45, *p* < .001], a difference that remained about the same for the rest of the period [quadratic contrast: χ^2^(1) = 11.53, *p* = .001]; see Figure 2 and Table 2. The young CG was not as successful, and no significant polynomial contrasts were found. In week 130, 88.6% of the IG and 69.7% of the CG participants were back at work to any extent, a difference that borders on statistical significance (*p* = .054, dichotomized data). As depicted in Figure 2, there were no signs of a converging RTW pattern between the younger IG and CG groups during the extended follow-up period.

**Table 2:** **Sick leave in the younger halves of the intervention and control groups as a function of weeks**

	Sick leave (%)					χ ^2^	***P***
	0	25	50	75	100		
	Week 0						
Control*	15.2 (5)	12.1 (4)	15.2 (5)	6.1 (2)	51.5 (17)	0.37	.985
IG**	11.4 (4)	11.4 (4)	17.1 (6)	8.6 (3)	51.4 (18)		
	Week 10						
Control	45.5 (15)	9.1 (3)	9.1 (3)	0	36.4 (12)	8.58	.035
IG	14.3 (5)	14.3 (5)	22.9 (8)	0	48.6 (17)		
	Week 20						
Control	63.6 (21)	3.0 (1)	3.0 (1)	0	30.3 (10)	14.22	.007
IG	28.6 (10)	8.6 (3)	28.6 (10)	5.7 (2)	28.6 (10)		
	Week 30						
Control	60.6 (20)	3.0 (1)	6.1 (2)	0	30.3 (10)	13.23	.010
IG	31.4 (11)	8.6 (3)	34.3 (12)	5.7 (2)	20.0 (7)		
	Week 40						
Control	66.7 (22)	3.0 (1)	3.0 (1)	0	27.3 (9)	7.77	.100
IG	42.9 (15)	8.6 (3)	20.0 (7)	2.9 (1)	25.7 (9)		
	Week 50						
Control	66.7 (22)	0	3.0 (1)	0	30.3 (10)	11.00	.027
IG	45.7 (16)	11.4 (4)	17.1 (6)	5.7 (2)	20.0 (7)		
	Week 60						
Control	66.7 (22)	3.0 (1)	3.0 (1)	0	27.3 (9)	5.91	.206
IG	60.0 (21)	8.6 (3)	11.4 (4)	5.7 (2)	14.3 (5)		
	Week 70						
Control	60.6 (20)	6.1 (2)	3.0 (1)	0	30.3 (10)	7.79	.099
IG	65.7 (23)	8.6 (3)	14.3 (5)	2.9 (1)	8.6 (3)		
	Week 80						
Control	63.6 (21)	0	9.1 (3)	0	27.3 (9)	6.44	.169
IG	71.4 (25)	5.7 (2)	11.4 (4)	2.9 (1)	8.6 (3)		
	Week 90						
Control	63.6 (21)	0	6.1 (2)	0	30.3 (10)	5.01	.286
IG	68.6 (24)	5.7 (2)	8.6 (3)	2.9 (1)	14.3 (5)		
	Week 100						
Control	63.6 (21)	3.0 (1)	6.1 (2)	0	27.3 (9)	4.68	.197
IG	74.3 (26)	8.6 (3)	8.6 (3)	0	8.6 (3)		
	Week 110						
Control	66.7 (22)	3.0 (1)	3.0 (1)	0	27.3 (9)	2.61	.456
IG	71.4 (25)	5.7 (2)	8.6 (3)	0	14.3 (5)		
	Week 120						
Control	63.6 (21)	3.0 (1)	3.0 (1)	0	30.3 (10)	4.38	.223
IG	74.3 (26)	5.7 (2)	8.6 (3)	0	20.6 (14)		
	Week 130						
Control	69.7 (23)	0	0	0	30.3 (10)	5.21	.157
IG	82.9 (29)	2.9 (1)	2.9 (1)	0	11.4 (4)		

*n = 35.

**n = 33.

The participants were 45 years or younger at their first day of sick leave. Figures show percentages within each group and the corresponding number of persons. Group comparisons with Pearson χ2-test were done on the full range of sick leave steps.

### Analyses of representativity and dropouts

An analysis of the 12 participants that had been excluded from the data set - due to retirement, denial of data use or matching problems - showed that 11 of the 12 drop-out participants belonged to the older part of the group (aged 46 or above at the beginning of sick-leave). The older drop-out subgroup consisted of 6 IG participants whom all had a RTW to some extent at the end of the previous follow-up (week 80), and 5 CG participants of whom 4 had RTW to some extent. The single drop-out in the younger subgroup was a CG participant on full-time sick leave at week 80.

At the beginning of the follow-up period (week 0) 57.4% of the IG participants and 51.5% of the CG participants in the somewhat smaller sample of participants with available data for the extended follow-up period (n = 68 pairs) had a RTW at any extent, again similar to the previous rates reported for the full sample (n = 74 pairs; 55.4% RTW in both groups; [8]). In the somewhat smaller sample 86.8% of IG participants and 75.0% of the CG participants had returned to work to any extent at the end of the former follow-up period (week 80), which are figures close to the ones previously reported for the original full sample of 74 pairs (89.2% vs. 73.0%; [8]).

### Relapses into increased degree of sick-leave

Analyzes of individual RTW profiles revealed that nine CG participants and four IG participants never returned to work during the entire 130-week follow-up period. Among the remaining participants, 49% of the CG participants and 52% of the IG participants had no relapse to a higher degree of sick-leave at a length of at least two weeks during the 130 weeks, while others had between 1 and 4 relapses. The overall distribution of relapses did not differ between IG and CG (*p* = .26), see Table 3.

**Table 3:** **Number of relapses into increased degree of sick leave in the intervention and control groups**

	Group		
Number of relapses	Intervention (n = 64)*	Control (n = 59)*	Comparison of distributions
None	51.6% (33)	49.2% (29)	χ^2^ = 5.52, p = .26
One	28.1% (18)	28.8% (17)	
Two	14.1% (9)	8.5% (5)	
Three	6.2% (4)	6.8% (4)	
Four	0	6.8% (4)	
Five or more	0	0	

*Nine controls and four intervention participants were excluded from the analysis because they never returned to work during the entire follow-up period.

A relapse was defined as a period of two consecutive weeks, or longer, with a higher degree of sick leave than the preceding week, during the total follow-up period of 130 weeks. Figures show percentages within each group and the corresponding number of persons.

### Initial part-time RTW as a predictor for later full-time RTW

A stable full-time RTW, defined as no sick leave during the final 26 weeks of the follow-up period, was observed among 79 participants (40 IG and 39 CG participants), of which 62% (30 IG and 19 CG participants) had been on partial sick-leave (25-75%) during at least 4 of the preceding 104 weeks. Of the 57 participants (28 IG and 29 CG participants) that failed to fulfill the criterion of stable full-time RTW, 60% (23 IG and 11 CG participants) had been on part time sick-leave during the same period. Thus, the implementation of partial sick leave was not more common among those with stable full-time RTW (62% vs. 60%; *p* = .78). When defining part-time sick leave as a more substantial part of the study period - during at least 26 of the initial 104 weeks - 27 (34%) of the participants with stable full-time RTW were found to have had such extended part-time sick-leave, compared with 23 (40%) of the participants failing to fulfil the stable full-time RTW criterion, a statistically non-significant difference (*p* = .46). For all participants who had been on part-time sick-leave (n = 95), the total number of weeks on part-time sick leave during the initial 104 weeks were differently distributed across subgroups; the group with a stable full-time RTW (n = 59) had had a median of 20 weeks on partial sick-leave, compared to a median of 41 weeks in the group failing to fulfil the stable full-time RTW criterion (n = 36; *p* = .002). The latter group also had a slightly higher degree of sick-leave during their part-time sick leave weeks (medians: 49% vs. 41% for the stable full-time RTW group; *p* = .03).

## Discussion

### Main findings

The main aim of the present study was to examine whether our previously shown successful RTW after a workplace-oriented intervention, for subjects on sick leave due to burnout, was sustained and stable or possibly increasing in a longer time perspective. A 50-week continuous prolongation of the original 80-week follow-up indicated that RTW in the intervention group (IG) as well as the control group (CG) was sustained and stable, though only in the younger half of the group.

Secondly, based on the long-term RTW improvements shown in the two Swedish studies with prolonged follow-up, we hypothesized that there may be a commonly occurring course in the direction toward RTW, where the speed of this course may depend in part on whether actions are taken to involve the workplace in facilitating RTW. Within the prolonged time frame used in the present study, no convergence between the two groups’ RTW rate was seen that could be taken as supporting such an assumption. The present study showed a significantly better RTW course in the IG than in the CG in the younger half of the group when analyzing the whole period from Week 0 to Week 130. In contrast, in the prolonged follow-up period alone (Week 81 to week 130), static patterns were seen, indicating a sustained difference in RTW between the two younger groups, while RTW was more or less similar in the older groups across the entire follow-up-period. Thus, no further improvements were seen during the extended approximately 12-month long follow-up.

A supplementary aim was to investigate whether relapses into increased degrees of sick leave were less frequent in the IG, due to the fact that the CDM often included a discussion about how to improve the patient’s and the supervisor’s preparedness for detecting early signs of a relapse. However, no difference in the extent of relapses between the IG and the CG were observed. Around 50% of each group had no relapse into increased sick leave during the total follow-up period and only around 1/5 of the participants in each group had two or more relapses.

Another supplementary aim was to examine the assumption that part-time work constitutes a pathway to full-time work [7]. However, the implementation of partial sick leave could not be shown to have occurred more frequently among those having attained a stable full-time RTW during the final 26 weeks of the follow-up period, irrespective of whether “part-time sick leave” was defined in terms of an extensive period (at least 26 weeks) or a brief period (at least 4 weeks) of the first 104 weeks of the follow-up. One possible explanation for this might be that the group failing to attain a stable full-time RTW towards the end of the follow-up period, had more severe burnout symptoms and needed a longer period of gradual RTW. Some support for this notion is found in the observations that the latter group showed a significantly longer duration of part-time sick leave and also had a higher degree of sick leave during the first 104 weeks.

### Comparison with other studies

The findings from the two other Swedish studies with long-term follow-ups showed improvements in RTW rates, though not as function of intervention [5, 15]. It should be kept in mind that Grossi’s and Santell’s [5] study was very small (n = 12 per group), which limits the ability to detect such differences. Stenlund’s [15] study had a response rate of 65% compared to their 1-year follow-up, which due to possible systematic differences between drop-outs and participants may introduce some uncertainty as regards interpreting the results, as discussed by the authors. Van Oostrom et al. [9] conducted a randomized control trial study of an intervention model that seems to share some characteristics with our model. In contrast to our results, they did not find any general superior effect on RTW of the intervention, but they did find an effect for those who reported being motivated to RTW despite symptoms [9]. Motivation to RTW was not measured in our study, and there were also a number of other differences, such as the time and reason for enrollment in the intervention, the selection of population, and the analysis strategies. Van Oostroms [9] study was carried out within one company, and included predominantly men with shorter sick leave periods for seemingly lighter forms of distress; thus the studies are not quite comparable. One strength of van Oostrom’s study [9], however, is that it was a randomized control trial, while randomization turned out not to be feasible in our study.

### Methodological issues

The present study has some limitations. One is the attrition pattern. An age effect was found, which is why the group was split into a younger and an older half. The drop-outs were found almost entirely in the older half, and among the remaining participants in the older age group the rates of RTW to any extent was very similar in the IG and the CG already immediately before the start of extended follow-up (week 80), with 82% and 77%, respectively. It is possible that a differential attrition occurred, since all older participants that dropped out from the IG had a RTW at week 80, while this was not the case with all CG drop-outs. Therefore, it is hard to draw any firm conclusions about RTW stability in the older half of the group. Moreover, a negative consequence of the division of the groups into age halves is a reduction in statistical power. Conversely, it could be argued that because the drop-outs were concentrated to a clearly distinguishable subgroup, detected by an interaction effect in the statistical analyses, the other half of the entire group was left almost intact. The younger group showed largely the same RTW pattern at week 80 as the entire group did at the original follow-up, suggesting that the younger group was fairly representative of the entire group at that time. This conclusion is supported by the lack of age effects on RTW in the original study.

One strength of the present study is that the recruitment base was an unselected sample of the population of the southern county of Sweden, probably leading to high generalizability of the results. Another strength is the detailed sick-listing register data retrieved from the RSIO, allowing us to conduct a continuous follow-up of the sick-listing process.

## Conclusions

It can be concluded that our previously reported good RTW results from a workplace-oriented intervention for patients on sick leave for burnout were stable over a long-term period only among participants below the age of 46. The absence of a converging RTW pattern between the intervention group and the controls indicates that the superior effect of the workplace-oriented intervention model among younger persons is not due to merely increasing the pace of the course of RTW. Occasional sick leave relapses were equally common in the intervention group and the control group. The assumption that part-time sick leave could predict a subsequent stable full-time RTW was not supported.
